# An Improved Sparrow Search Algorithm and Its Application in HIFU Sound Field

**DOI:** 10.1155/2023/1228685

**Published:** 2023-03-03

**Authors:** Yihao Yang, Jinxu Tao, Jiayu Zhou, Jiaqi Wang, Xinyu Guo

**Affiliations:** Department of Electronic Engineering and Information, University of Science and Technology of China, Hefei, Anhui 230026, China

## Abstract

The sparrow search algorithm (SSA) is a novel swarm intelligence optimization algorithm. It has a fast convergence speed and strong global search ability. However, SSA also has many shortcomings, such as the unstable quality of the initial population, easy to fall into the local optimal solution, and the diversity of the population decreases with the iterative process. In order to solve these problems, this paper proposes an improved sparrow search algorithm (ISSA). ISSA uses Chebyshev chaotic map and elite opposition-based learning strategy to initialize the population and improve the quality of the initial population. In the process of producer location update, dynamic weight factor and Levy flight strategy are introduced to avoid falling into a local optimal solution. The mutation strategy is applied to the scrounger location update process, and the mutation operation is performed on individuals to increase the diversity of the population. In order to verify the feasibility and effectiveness of ISSA, it is tested on 23 benchmark functions. The results show that compared with other seven algorithms, ISSA has higher convergence accuracy, faster convergence speed, and stronger stability. Finally, ISSA is used to optimize the sound field of high-intensity focused ultrasound (HIFU). The results show that ISSA can effectively improve the focusing performance and reduce the influence of sound field sidelobe, which is of great benefit for HIFU treatment.

## 1. Introduction

Optimization methods are widely used in many fields, such as signal processing [1], image processing [2], and machine learning [3]. However, a large number of problems encountered in real life are very complex, and it is difficult to find the global optimal solution. The meta-heuristic algorithm has attracted the attention of researchers because of its simplicity, easy implementation, independent of specific problems, and avoiding falling into local optimal solutions. Classic meta-heuristic algorithms include the genetic algorithm (GA) [4], particle swarm optimization (PSO) [5], grey wolf optimizer (GWO) [6], and whale optimization algorithm (WOA) [7]. These algorithms have been applied to many optimization problems and show excellent performance. In recent years, more and more meta-heuristic algorithms have been proposed, such as the moth search algorithm (MSA) [8], harris hawks optimization (HHO) [9], sparrow search algorithm (SSA) [10], slime mould algorithm (SMA) [11], social network search (SNS) [12], and fusion-fission optimization (FuFiO) [13].

The sparrow search algorithm (SSA) is a swarm intelligence optimization algorithm proposed by Xue and Shen in 2020 and inspired by foraging and antipredation behaviors of sparrows [10]. It has been proved that SSA has faster convergence speed and better performance than the classical meta-heuristic algorithms PSO and GWO [10]. Among the meta-heuristic algorithms proposed in recent years, SSA has received high attention and has been applied to many types of engineering problems [14–16]. Therefore, we choose SSA for research. Compared with other algorithms, SSA has some advantages in convergence speed and global search ability. Nevertheless, when solving complex problems, the performance of SSA is greatly affected by the initial population, and the diversity of the population will decrease significantly with the iterative process [17]. In addition, in the optimization process, the convergence accuracy of SSA needs to be improved, and the ability to jump out of the local optimal solution needs to be enhanced.

Many scholars have made improvements to SSA. Lyu et al. [18] use the chaotic map to initialize the population, which ensures the quality of the initial solution and improves the diversity of the initial population. However, this method is stochastic and does not make full use of the information carried by high-quality individuals in the initial population. Song et al. [19] introduce nonlinear decreasing weight to improve the ability of global exploration and local exploitation, but this method cannot improve the ability to jump out of the local optimal solution. Zhang et al. [20] combine the sine cosine algorithm with SSA to help SSA jump out of the local optimal solution, but this method is stochastic, and if the solution space is not well selected, it still cannot jump out of the local optimal solution.

In order to overcome the above shortcomings of SSA, an improved sparrow search algorithm (ISSA) is proposed in this paper. In the initial population stage, ISSA uses Chebyshev chaotic map to improve the diversity of the population and uses an elite opposition-based learning strategy to produce a high-quality population. When updating the producer's location, the dynamic weight factor is introduced to balance the producer's ability of global exploration and local exploitation, and the Levy flight strategy is used to expand the search space to avoid falling into the local optimal solution. The mutation strategy is used to update the scrounger's position, guide individuals to approach the optimal solution, and improve population diversity and global search ability.

High-intensity focused ultrasound (HIFU) is a high technology for the treatment of tumors. HIFU has been initially applied to the clinical treatment of soft tissue tumors such as breast cancer and uterine leiomyoma by virtue of its advantages of minimally invasive and noninvasive, less complications, and repeatable treatment [21]. The principle of HIFU treatment can be simply summarized as follows: the low-energy ultrasound emitted by each array element of focused ultrasound transducer passes through skin, blood, bone, and other tissues and converges in the target area. Under the thermal, mechanical, and cavitation effects of ultrasound, the tumor tissue in the target area heats up rapidly, and thermal coagulation necrosis occurs, thus losing the ability of proliferation, infiltration, and metastasis [22]. The therapeutic effect of HIFU depends on the focusing accuracy and temperature. In HIFU sound field, the existence of acoustic sidelobe will reduce the focusing performance.

Studies by many scholars have shown that by optimizing the sound field of focused ultrasound and suppressing the acoustic sidelobe, the focusing performance and the therapeutic effect of focused ultrasound can be improved. Wang et al. [23] proposed an objective function for optimizing the sound field, but the problem of the maximum or minimum value of the objective function was not solved. Therefore, this paper uses ISSA to solve the maximum value of the objective function to optimize HIFU sound field. The main contributions of this paper are as follows:

- SSA is improved from the perspective of elite individuals, initial population, and search space
- ISSA is verified by Wilcoxon's rank-sum test and time complexity analysis
- ISSA is used to optimize the HIFU sound field and suppress the acoustic sidelobe

The rest of this paper is organized as follows.  Section 2 outlines the key steps of SSA.  Section 3 introduces the proposed ISSA in detail.  Section 4 describes the HIFU sound field models.  Section 5 introduces the simulation experiment and results.  Section 6 summarizes the work of this paper and points out the next research direction.

## 2. Model of the SSA

The SSA is a swarm intelligence optimization algorithm based on foraging and antipredation behaviors of sparrows. In SSA, individuals in sparrow population are divided into three different types: producer, scrounger, and scouter. The producers have high energy reserves, strong exploration ability, and broad exploration space and are responsible for finding foraging areas with rich food for the whole population. When the sparrow detects the predator, the producers need to lead other individuals to a safe area to avoid the predator's attack. The location update equation of the producers is as follows:

(1) $$\begin{array}{c} {X_{i,j}}^{t+1}=\left\{\begin{array}{cc} {X_{i,j}}^{t}\cdot \exp\left(\frac{-i}{\alpha \cdot T_{\max}}\right)\text{,} & R_{2}<ST\text{,}\\ {X_{i,j}}^{t}+Q\cdot L\text{,} & R_{2}\geq ST\text{,} \end{array}\right. \end{array}$$

where *t* represents the current number of iterations, *X*_*i*,*j*_ represents the position of the ith sparrow on the jth dimension (*j*=1, 2,…, dim), *α* ∈ (0, 1] is a random number, *T*_max_ represents the maximum number of iterations, *R*_2_ ∈ [0, 1] and ST ∈ [0.5, 1] represent the alarm value and safety threshold, respectively, *Q* is a random number obeying the normal distribution, *L* is a 1 × dim row vector, and all elements in it are 1.

The scroungers always follow the producers to obtain high-quality food and increase their energy reserves. Some scroungers monitor the producers and compete with them for food. When the energy reserve of the scroungers is low, they will fly away from the population and look for food by themselves to survive. The location update equation of the scroungers is as follows:

(2) $$\begin{array}{c} {X_{i,j}}^{t+1}=\left\{\begin{array}{cc} Q\cdot \exp\left(\frac{{X_{worst}}^{t}-{X_{i,j}}^{t}}{i^{2}}\right)\text{,} & i>n/2\text{,}\\ {X_{P}}^{t+1}+\left|{X_{i,j}}^{t}-{X_{P}}^{t+1}\right|\cdot A^{+}\cdot L\text{,} & otherwise\text{,} \end{array}\right. \end{array}$$

where *X*_worst_^*t*^ is the current global worst position, *n* is the number of individuals in the population, *X*_*P*_^*t*+1^ is the global best position found by the producers, *A* is a 1 × dim row vector, the elements in it are randomly assigned 1 or −1, and *A*^+^=*A*^*T*^(AA^*T*^)^−1^ represents the MP inverse of *A*.

In the sparrow population, some individuals play the role of the scouter. These individuals can detect the threat posed by predators and send out alerts to other individuals to avoid. In the simulation experiment, it is assumed that such individuals account for 10% to 20% of the total population, and their initial positions are randomly assigned. The location update equation of the scouters is as follows:

(3) $$\begin{array}{c} {X_{i,j}}^{t+1}=\left\{\begin{array}{cc} {X_{best}}^{t}+\beta \cdot \left|{X_{i,j}}^{t}-{X_{best}}^{t}\right|\text{,} & f_{i}>f_{g}\text{,}\\ {X_{i,j}}^{t}+K\cdot \left(\frac{\left|{X_{i,j}}^{t}-{X_{worst}}^{t}\right|}{\left(f_{i}-f_{w}\right)+\varepsilon }\right)\text{,} & f_{i}=f_{g}\text{,} \end{array}\right. \end{array}$$

where *X*_best_^*t*^ is the current global optimal location; *β*, as the step size control factor, is a random number that obeys the normal distribution with mean value of 0 and variance of 1; *K* ∈ [−1, 1] is a random number; *f*_*i*_ is the fitness value of the current individual (objective function value); *f*_*g*_ and *f*_*w*_ represent the current global optimal and worst fitness values, respectively; *ε* is a very small number so as to avoid denominator being 0.

## 3. Model of the ISSA

### 3.1. Chebyshev Chaotic Map and Elite Opposition-Based Learning Strategy

In swarm intelligence optimization algorithm, the quality of initial population directly affects the convergence performance of the algorithm. In SSA, the initial population is generated randomly, which makes the distribution of the initial population uneven and the quality unstable, and reduces the convergence accuracy and convergence speed. Chaotic mapping has the characteristics of randomness, ergodicity, and regularity. In recent years, it has been used in swarm intelligence algorithm to improve the quality of the initial population. Commonly used chaotic maps include Tent chaotic map [24], Kent chaotic map [25], and Logistic chaotic map [26]. In this paper, Chebyshev chaotic map is used to initialize the population. Compared with the above chaotic mapping, Chebyshev chaotic map is simpler, insensitive to the initial value, and the mapping results are more evenly distributed. Chebyshev chaotic map equation is as follows:

(4) $$\begin{array}{c} x^{t+1}=\cos\;\left(t\;\cos^{-1}\left(x^{t}\right)\right)\text{,} \end{array}$$

where *x*^1^ ∈ [0, 1] is a random number. After obtaining the Chebyshev chaotic sequence, the initial population is generated by the following equation:

(5) $$\begin{array}{c} {X_{i,j}}^{t+1}={lb}_{j}+\left({ub}_{j}-{lb}_{j}\right)\times x^{t}\text{,} \end{array}$$

where *lb*_*j*_ and *ub*_*j*_ represent the lower and upper boundary of the jth dimension of the search space, respectively.

The elite opposition-based learning strategy (EOLS) is used to improve the quality of the initial population [27]. In the sparrow population, there are some elite individuals. Whether it is the ability to search or resist the enemy, elite individuals are better than other individuals. The basic idea of the EOLS is to use the information carried by elite individuals as much as possible to generate the initial population, so as to improve the quality of the population, enrich the diversity of the population, and avoid the algorithm falling into the local optimal solution.

Generally speaking, the elite individuals are individuals with small fitness value in the population. After obtaining the initial population, the individuals are sorted according to the fitness value, and several individuals with small fitness value are selected to form the elite group. For each elite individual in the elite group, its elite opposition can be calculated by the following equation:

(6) $$\begin{array}{c} \tilde{X_{i,j}}=\mu \left(\tilde{{lb}_{j}}+\tilde{{ub}_{j}}\right)-X_{i,j}\text{,} \end{array}$$

where *μ* ∈ [0, 1] is a random number, and $\tilde{{lb}_{j}}$ and $\tilde{{ub}_{j}}$ represents the lower and upper boundary of the individual in the initial population in the jth dimension of the current search space, respectively. After the elite opposition set is obtained by (6), the initial population is combined with the set, the fitness values of all individuals are calculated again, and *n* individuals with small fitness values are selected to form the real initial population.

### 3.2. Dynamic Weight Factor and Levy Flight Strategy

In the sparrow population, the producers are responsible for exploring and exploiting the search space and looking for areas with rich food resources. Therefore, the producers need to adopt flexible strategies to balance the ability of global exploration and local exploitation. In SSA, it can be seen from (1) that the position update weight of the producers is unchanged. In the later stage of iteration, the producers still use a large step for exploitation, which greatly reduces the ability of local exploitation. This paper solves this problem by introducing dynamic weight factor, which is expressed as follows:

(7) $$\begin{array}{c} {X_{i,j}}^{t+1}=\left\{\begin{array}{cc} \omega \cdot {X_{i,j}}^{t}\cdot \exp\left(\frac{-i}{\alpha \cdot T_{\max}}\right)\text{,} & R_{2}<ST\text{,}\\ \omega \cdot {X_{i,j}}^{t}+Q\cdot L\text{,} & R_{2}\geq ST\text{,} \end{array}\right. \end{array}$$

(8) $$\begin{array}{c} \omega ={\left(\frac{T_{\max}-t+1}{T_{\max}}\right)}^{t}+\delta \text{,} \end{array}$$

where *δ* ∈ [0, 0.1] is a random number, which is used to avoid the dynamic weight factor *ω* being too small in the later stage of iteration. It can be seen from (8) that the dynamic weight factor *ω* is large at the beginning of the iteration but decreases sharply with the iterative process. Dynamic weight factor *ω* ensures that the producers can perform global exploration with a larger step size at the initial stage of the iteration and perform local exploitation with a smaller step size at the later stage of the iteration, which balances the ability of global exploration and local exploitation.

If the producers have fallen into the local optimal solution in the early stage of iteration, exploitation can only be performed near the local optimal solution in the later stage of iteration. In order to avoid such a situation, this paper adopts the Levy flight strategy to help the producers still have the opportunity to jump out of the local optimal solution in the later stage of iteration. Levy flight is a non-Gaussian random process, and its step size obeys Levy distribution. It is very difficult to calculate the step size of Levy flight, so the Mantegna algorithm [28] is often used to simulate, and its expression is as follows:

(9) $$\begin{array}{c} s=\frac{u}{{\left|v\right|}^{1/\beta }}\text{,} \end{array}$$

where *u* ~ *N*(0, *σ*_*u*_^2^), *v* ~ *N*(0, *σ*_*v*_^2^), *σ*_*u*_ and *σ*_*v*_ is defined as

(10) $$\begin{array}{c} \sigma_{u}={\left[\frac{\Gamma \left(1+\beta \right)\sin\;\left(\pi \beta /2\right)}{\Gamma \left(\left(1+\beta \right)/2\right)2^{\left(\beta -1\right)/2}}\right]}^{1/\beta }\text{,}\\ \sigma_{v}=1\text{,} \end{array}$$

where Γ is the standard gamma function. *β* ∈ (0, 2) is a random number.

After obtaining the step *s* of Levy flight, the position of the producers is updated according to the following equation:

(11) $$\begin{array}{c} \tilde{{X_{i}}^{t}}={X_{i}}^{t}+0.01s\left({X_{i}}^{t}-{X_{b}}^{t}\right)\text{,} \end{array}$$

where *X*_*i*_^*t*^ is the location of the producers calculated by (7). *X*_*b*_^*t*^ is the current global optimal location. According to the characteristics of Levy distribution, Levy flight has many small steps, which can enhance the local exploitation ability of the producers. Occasionally, there are large steps to help the producers jump out of the local optimal solution and enhance the global exploration ability of the producers. The dynamic weight factor and Levy flight strategy complement each other, improve the efficiency of the producers, reduce the possibility of the producers falling into the local optimal solution, and better balance the ability of local exploitation and global exploration.

### 3.3. Mutation Strategy

In the sparrow population, the scroungers will monitor the behavior of the producers. When the producers find food, they compete with them to improve their energy reserves. Some of the scroungers with low energy reserves will fly away from the population and look for foraging areas alone. In SSA, the direction of the scroungers flying away from the population is determined by *X*_worst_^*t*^ and *X*_*i*,*j*_^*t*^ (*i* > *n*/2 in (2)). This update method cannot ensure that the scroungers find areas with rich food. In this paper, the mutation strategy [29] shown in the following is used to guide the flight of the scroungers and improve the diversity of the population.

(12) $$\begin{array}{c} {X_{i,j}}^{t+1}={X_{i,j}}^{t}+\eta \left({X_{P}}^{t+1}-{X_{i,j}}^{t}\right)\text{,} \end{array}$$

where *η* ∈ [0, 1] is a random number. The previous formula will guide the scroungers to the global optimal position *X*_*P*_^*t*+1^ and improve the probability of the scroungers finding high-quality food. In the simulation experiment, when *i* > *n*/2, the first equation in equations (2) and (12) is randomly selected to update the position of the scroungers to improve the diversity of the population. The implementation steps of ISSA are shown in Algorithm 1.

## 4. Model of the HIFU Sound Field

**Figure 1:**
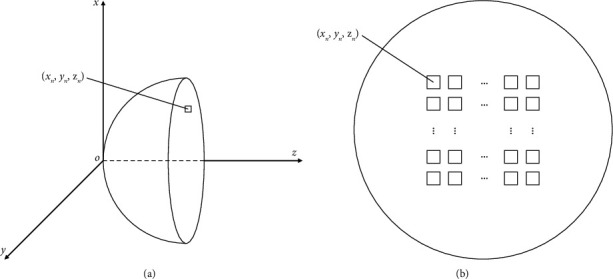
Concave spherical phased array transducer model: (a) side view and (b) main view.

Common focused ultrasonic transducers can be divided into three types: concave spherical self-focusing transducer, acoustic lens focusing transducer, and phased array focusing transducer. The concave spherical self-focusing transducer adjusts the focusing position by changing the size and curvature of the concave spherical surface. Therefore, the focus of this transducer is fixed, and the position of the focus can only be changed by moving the transducer. The acoustic lens focusing transducer uses the lens to converge the sound wave to the target area. The reflection and refraction of sound waves through the lens will lose part of the energy, and the lens itself will absorb the energy of sound waves to generate heat. Therefore, it is necessary to select materials with low loss and high temperature resistance. The phased array focusing transducer generates a sound wave with a certain amplitude and phase by controlling each array element and realizes one-point or multipoint focusing according to the principle of wave interference. Compared with the above two kinds of transducers, the focusing position and depth of phased array transducer are adjustable, and the precision is higher. Therefore, the concave spherical phased array transducer shown in Figure 1 is selected for simulation in this paper.

In the simulation experiment, 256 rectangular array elements (shown in the rectangular box in Figure 1) are evenly arranged inside the concave sphere. The sound pressure at any point in the sound field generated by the concave spherical phased array [30] is given by the following equation:

(13) $$\begin{array}{c} p\left(x,y,z\right)=\frac{j\rho c}{\lambda }\overset{N}{\underset{n=1}{\sum }}u_{n}\frac{F_{n}∆w∆h}{R_{n}}e^{-\left(\alpha +jk\right)\tilde{R_{SR}}}sinc\frac{k\tilde{x_{n}}∆w}{2R}sinc\frac{k\tilde{y_{n}}∆h}{2R}\text{,} \end{array}$$

where $j=\sqrt{-1}$ represents imaginary unit. *ρ* represents the density of the medium. *λ* represents the wavelength of the sound wave. *c* represents the velocity of the sound wave in the medium. *u*_*n*_ represents the vibration velocity of a particle perpendicular to the surface of the array element. ∆*w* and ∆*h* represents the length and width of the array element respectively. *α* represents the attenuation coefficient of sound wave. *k* represents the wave number. *R* represents the distance from any point (*x*, *y*, *z*) to the projection point of the array element center (*x*_*n*_, *y*_*n*_, *z*_*n*_) in the *xy* plane. For the calculation of other parameters, please refer to reference [30].

Equation (13) can be expressed as a matrix as follows:

(14) $$\begin{array}{c} P=H_{M}\cdot u_{N}\text{,} \end{array}$$

*M* represents the number of focal points. *N* represents the number of array elements. *u*_*N*_ represents the array element driving signal vector

(15) $$\begin{array}{c} u_{N}={\left[u_{1},u_{2},\ldots ,u_{N}\right]}^{T}\text{,} \end{array}$$

 *H*_*M*_ represents the forward transmission operator of sound field, which is an *M* × *N* matrix

(16) $$\begin{array}{c} H_{M}=\left[\begin{array}{cc} \begin{array}{cc} h_{11} & h_{12}\\ h_{21} & h_{22} \end{array} & \begin{array}{cc} \cdots & h_{1N}\\ \cdots & h_{2N} \end{array}\\ \begin{array}{cc} \vdots & \vdots \\ h_{M1} & h_{M2} \end{array} & \begin{array}{cc} \ddots & \vdots \\ \cdots & h_{MN} \end{array} \end{array}\right]\text{,} \end{array}$$

*P* represents the sound pressure vector of the focal point

(17) $$\begin{array}{c} P={\left[p_{1},p_{2},\ldots ,p_{M}\right]}^{T}\text{.} \end{array}$$

We can set the sound pressure vector of the focal point *P* to the desired value. Then, the array element driving signal vector *u*_*N*_ can be calculated by the following equation:

(18) $$\begin{array}{c} u_{N}={H_{M}}^{H}\cdot {\left(H_{M}{H_{M}}^{H}\right)}^{-1}\cdot P\text{.} \end{array}$$

After obtaining *u*_*N*_, the sound pressure at any point in the sound field can be calculated by equation (13).

The above method only specifies the sound pressure value *p*_*i*_(*i*=1,2,…, *M*) of each focal point, but does not specify the phase, so each focal point is formed at the same time. In fact, by adjusting the phase of each focal point, not only focusing can be achieved, but the optimal focusing effect can also be obtained. Rewrite the sound pressure vector *P* as

(19) $$\begin{array}{c} P={\left[p_{1}e^{j\theta_{1}},p_{2}e^{j\theta_{2}},\ldots ,p_{M}e^{j\theta_{M}}\right]}^{T}\text{.} \end{array}$$

Optimal focusing effect is achieved by maximizing the sound pressure gain function

(20) $$\begin{array}{c} \max\frac{P^{H}P}{P^{H}{\left(H_{M}{H_{M}}^{H}\right)}^{-1}P}\text{.} \end{array}$$

## 5. Experiments and Results

All simulation experiments are performed on an Intel Core i7-11800H CPU @2.30GHz. All codes are implemented on MATLAB R2020b.

### 5.1. Algorithm Performance Test

In the simulation experiments, the performances of the ISSA, GA, PSO, GWO, WOA, HHO, SSA, and SNS on 23 benchmark functions are compared to verify the feasibility and effectiveness of ISSA. These 23 benchmark functions are divided into three categories: unimodal functions, multimodal functions (both of which have a dimension of 30), and fixed-dimension functions. The details are shown in Tables 1–3.

When comparing the performance of the eight algorithms, in order to ensure the fairness and objectivity of the results, the same values are set for the common parameters: the population size *n* is set to 100, and the maximum number of iterations *T*_max_ is set to 500. In ISSA and SSA, the proportion of the producers and the scroungers is set to 20% and 80%, respectively, the proportion of the scouters is set to 20%, and the safety threshold *ST* is set to 0.8. In GA, the crossover probability *p*_*c*_ and mutation probability *p*_*m*_ adaptively change. In PSO, learning factor *c*_1_=*c*_2_=1.5, inertia weight *w*_max_=0.8, *w*_min_=0.4, speed *v*_max_=1, *v*_min_=−1.

**Figure 2:**
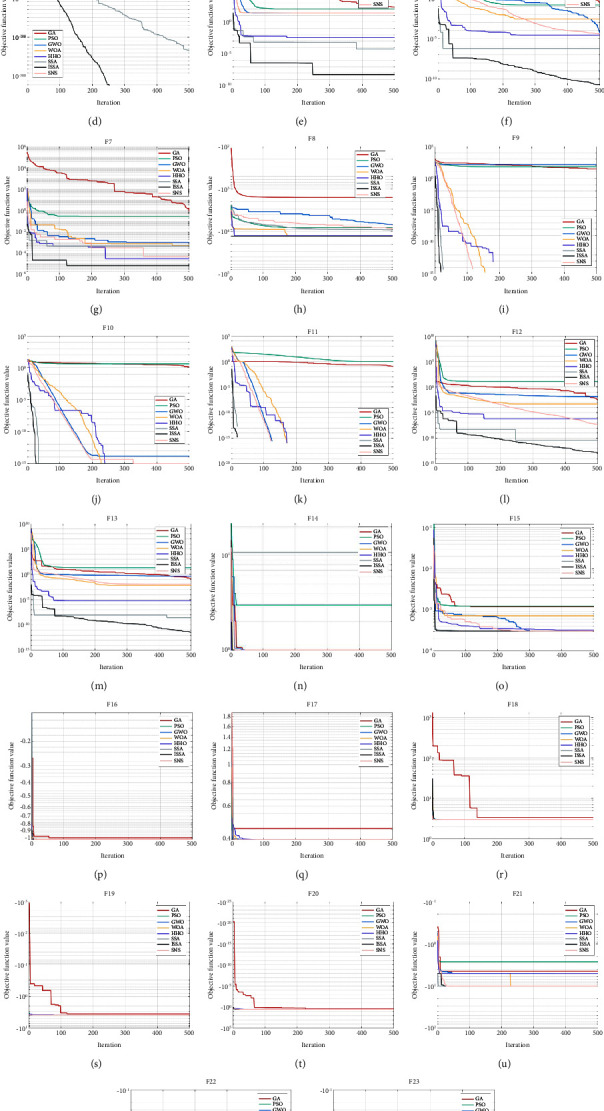
Convergence curves of eight algorithms on benchmark functions. (a) F1. (b) F2. (c) F3. (d) F4. (e) F5. (f) F6. (g) F7. (h) F8. (i) F9. (j) F10. (k) F11. (l) F12. (m) F13. (n) F14. (o) F15. (p) F16. (q) F17. (r) F18. (s) F19. (t) F20. (u) F21. (v) F22. (w) F23.

30 experiments are conducted independently on each benchmark function, and the convergence curve of each algorithm is drawn. The results are shown in Figure 2. The minimum value, average value, and standard deviation of each algorithm are recorded, and the results are shown in Table 4. For the same benchmark function, the average value represents the convergence accuracy of the algorithm, and the standard deviation represents the stability of the algorithm.

For unimodal functions, i.e., F1 to F7, ISSA is superior to SSA in all indicators. For F1 to F4, ISSA can accurately find the optimal value of zero, and the average and standard deviation are also zero, indicating that the convergence accuracy and stability of ISSA are excellent. For F2 to F4, although SSA can also find the optimal value of zero, this does not mean that SSA can find the optimal value every time, because the average is not zero. The other six algorithms fail to find the optimal value of zero. For F5 to F7, ISSA and SSA do not converge to the global optimal solution, but ISSA converges faster. From the data in Table 4, it can be found that on F6, the convergence accuracy and stability of ISSA are at least two orders of magnitude higher than those of SSA, indicating that ISSA has higher convergence accuracy and better stability. On F5 and F7, the stability of ISSA is similar to that of SSA, but the convergence accuracy is at least two orders of magnitude higher than that of SSA, indicating that when the stability is similar, the convergence accuracy of ISSA is higher.

For multimodal functions, i.e., F8 to F13, ISSA is superior to SSA in most indicators. On F8, ISSA is not as stable as SSA, but the convergence accuracy is one order of magnitude higher than SSA, and the convergence speed is also much faster than SSA. For F9 to F11, the performance of ISSA is similar to that of SSA. On both F12 and F13, ISSA is two to three orders of magnitude higher than SSA, both in terms of convergence accuracy and stability. For the other six algorithms, except that the GWO, WOA, HHO, and SNS perform slightly worse than ISSA, both the GA and PSO are far inferior to ISSA.

For fixed dimension functions, i.e., F14 to F23, due to the low dimension, the indicators of ISSA and SSA are relatively close. On F15 to F20, except GA, the other seven algorithms can find or approach the optimal value, but ISSA is always the most stable. On F14, SNS has the best convergence accuracy and stability, while SSA has the worst convergence accuracy and stability. On F21, SNS has the best convergence accuracy and stability. On F22 and F23, ISSA has excellent convergence accuracy and stability.

### 5.2. Effectiveness Analysis of Improvement Strategies

**Figure 3:**
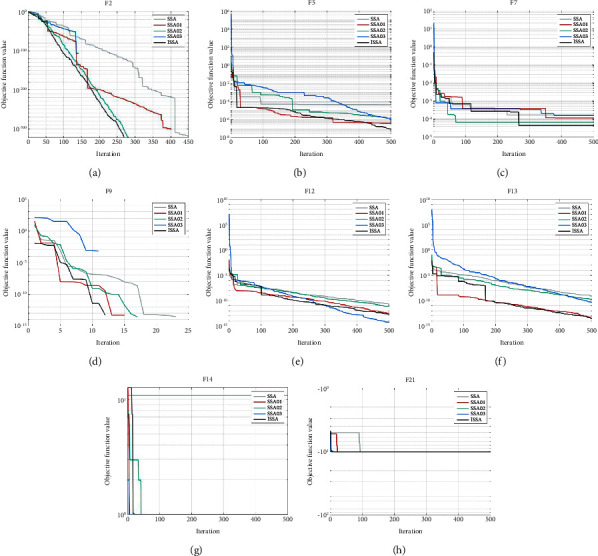
Convergence curves of different improvement strategies on benchmark functions: (a) F2, (b) F5, (c) F7, (d) F9, (e) F12, (f) F13, (g) F14, and (h) F21.

Based on the three strategies proposed in Section 3, this paper improves the convergence accuracy of SSA and enhances the convergence stability of SSA. But it is unclear whether all three strategies worked, so verification is needed. In order to compare the impact of different improvement strategies on the performance of the algorithm, SSA that only uses the Chebyshev chaotic map and elite opposition-based learning strategy (SSA01), SSA that only adopts the dynamic weight factor and Levy flight strategy (SSA02), SSA that only uses mutation strategy (SSA03), and ISSA are compared on eight test functions. The experimental results are shown in Figure 3 and Table 5.

As shown in Figure 3, SSA01, SSA02, and SSA03 converge faster than SSA, while the convergence speed of ISSA based on the three strategies is significantly improved. On F5, F7, and F13, although ISSA did not converge to the theoretical optimal value, the convergence speed and convergence accuracy are significantly better than the other four algorithms. On F2, all five algorithms converge to the theoretical optimal value, but with the same number of iterations, ISSA has higher convergence accuracy; with the same convergence accuracy, ISSA has a faster convergence speed. On the eight test functions, the convergence speed and convergence accuracy of SSA01, SSA02, and SSA03 are better than SSA, but slightly inferior to ISSA, indicating that each strategy has worked, and each strategy is very effective.

It can be seen from Table 5 that the convergence accuracy and stability of SSA01, SSA02, and SSA03 are better than those of SSA on most test functions, and the convergence accuracy and stability of ISSA are also significantly improved. On F5, F7, F12, and F14, although ISSA does not converge to the theoretical optimal value, both the convergence accuracy and the stability of the algorithm are better than SSA01, SSA02, and SSA03, indicating that under the joint influence of the three strategies, the convergence accuracy and stability of ISSA are both improved to the greatest extent.

SSA01 adopts the Chebyshev chaotic map to improve the diversity of the population and uses elite opposition-based learning strategy to generate high-quality populations. SSA02 introduces a dynamic weight factor to balance the ability of global exploration and local exploitation and uses the Levy flight strategy to expand the search space, avoid falling into the local optimal solution, and improve the convergence accuracy. SSA03 uses a mutation strategy to perform mutation operations on individuals to increase the diversity of the population and improve the ability to jump out of local optimal solutions. This further explains the feasibility of three strategies adopted in this paper.

### 5.3. Wilcoxon's Rank-Sum Test

Derrac et al. [31] suggest that statistical tests should be used when evaluating the performance of an algorithm. It is not sufficient to evaluate the performance of the algorithm only by the average and standard deviation, and other statistical tests should also be considered to demonstrate that the proposed improved algorithm has significant improvement over existing algorithms. In this paper, the Wilcoxon rank-sum test is used to further illustrate that the performance of ISSA is indeed significantly improved compared with other algorithms. Select the null hypothesis H0: the performance of two algorithms is similar, and the alternative hypothesis H1: the performance of two algorithms is significantly different. The test result *p* is used to compare the differences between the two algorithms. When *p* < 0.05, H0 is rejected, indicating that there is a significant difference in performance between the two algorithms. When *p* > 0.05, H0 is accepted; that is, the two algorithms have the same global optimization performance.

Table 6 shows the test results of ISSA and the other seven algorithms on 23 benchmark functions. *R* is the significance evaluation result: “+,” “−,” and “ = ,” respectively, represent the performance of ISSA is superior, inferior, and equivalent to the algorithms under comparison. NAN means that it cannot be compared; that is, the two algorithms under comparison both find the global optimal solution and cannot make a significant difference judgment.

It can be seen from Table 6 that only the *p* values of ISSA and SSA on F3 are slightly greater than 0.05, and the other *p* values are much less than 0.05. This indicates that the performance of ISSA and SSA on F3 is similar, while on other benchmark functions, the performance of ISSA is significantly different from the other seven algorithms. The *p* values of ISSA and SSA on F1, F9 to F11, F17, and F19 are NAN because both algorithms find the global optimal solution. The results of the Wilcoxon rank-sum test further illustrate that the performance of ISSA is indeed significantly improved compared with other algorithms.

### 5.4. Time Complexity Analysis

Suppose the number of individuals in the sparrow population is *N*, the dimension of solution space is *D*, and the maximum number of iterations is *T*_max_. Suppose the time required for initializing population parameters is *t*_0_, the time required for generating random numbers in each dimension is *t*_1_, the time for solving fitness function is *f*(*D*), and the time for sorting sparrows by fitness value is *t*_2_, and then, the time complexity of SSA in initializing population stage is

(21) $$\begin{array}{c} T_{0}=O\left[t_{0}+N\left(t_{1}D+f\left(D\right)\right)+t2\right]=O\left[D+f\left(D\right)\right]\text{.} \end{array}$$

When updating the location of producers, suppose the number of producers is *PD*, the time required to update the position of each dimension according to (1) is *t*_3_, the time required to generate random numbers *Q* and *α* is *t*_4_, and the time to solve the fitness function is *f*(*D*). The time complexity of this stage is

(22) $$\begin{array}{c} T_{1}=O\left[PD\cdot \left(\left(t_{3}+t_{4}+t_{4}\right)D+f\left(D\right)\right)\right]=O\left[D+f\left(D\right)\right]\text{.} \end{array}$$

When updating the position of scroungers, the number of scroungers is (*N* − *PD*), the time required to update the position of each dimension according to (2) is *t*_5_, the time to generate the random number *Q* is still *t*_4_, and the time to solve the fitness function is *f*(*D*). The time complexity of this stage is

(23) $$\begin{array}{c} T_{2}=O\left[\left(N-PD\right)\left(\left(t_{5}+t_{4}\right)D+f\left(D\right)\right)\right]=O\left[D+f\left(D\right)\right]\text{.} \end{array}$$

When updating the position of scouters, suppose the number of scouters is *SD*, the time required to update the position of each dimension according to (3) is *t*_6_, the time to generate random number *β* and *K* is *t*_7_, and the time to solve the fitness function is *f*(*D*). The time complexity of this stage is

(24) $$\begin{array}{c} T_{3}=O\left[SD\cdot \left(\left(t_{6}+t_{7}+t_{7}\right)D+f\left(D\right)\right)\right]=O\left[D+f\left(D\right)\right]\text{.} \end{array}$$

To sum up, the time complexity of SSA is

(25) $$\begin{array}{c} T=T_{0}+T_{\max}\left(T_{1}+T_{2}+T_{3}\right)=O\left[D+f\left(D\right)\right]\text{.} \end{array}$$

Now, the time complexity of ISSA is analyzed. Suppose the time required for initializing population parameters is *η*_0_, the time required to initialize the position of each dimension according to (4) and (5) is *η*_1_, and the time to solve the fitness function is *f*(*D*). Suppose the proportion of elite individuals is *r*, then the number of elite individuals is *rN*. Suppose the time required to generate the elite individual position of each dimension according to (6) is *η*_2_, the time to solve the fitness function is still *f*(*D*), and the time to sort and generate the real initial population is *η*_3_. Then, the time complexity of this stage is

(26) $$\begin{array}{c} {T_{0}}^{\prime }=O\left[\eta_{0}+N\left(\eta_{1}D+f\left(D\right)\right)+rN\left(\eta_{2}D+f\left(D\right)\right)+\eta_{3}\right]=O\left[D+f\left(D\right)\right]\text{.} \end{array}$$

When updating the position of producers, suppose the time required to generate the dynamic weight factor according to (8) is *η*_4_, the time required to update the position of each dimension according to (7) is *η*_5_, and the time to generate random numbers *Q* and *α* is *η*_6_. Suppose the time required to generate the Levy step according to (9) is *η*_7_, the time required to update the position of each dimension according to equation (12) is *η*_8_, and the time to solve the fitness function is *f*(*D*). Then, the time complexity of this stage is

(27) $$\begin{array}{c} {T_{1}}^{\prime }=O\left[\eta_{4}+PD\cdot \left(\left(\eta_{5}+\eta_{6}+\eta_{6}\right)D+\eta_{7}+\eta_{8}D+f\left(D\right)\right)\right]=O\left[D+f\left(D\right)\right]\text{.} \end{array}$$

When updating the position of scroungers, the time required to update the position of each dimension according to (2) or (12) is *η*_9_, the time to generate the random number *Q* is still *η*_6_, and the time to solve the fitness function is *f*(*D*). Then, the time complexity of this stage is

(28) $$\begin{array}{c} {T_{2}}^{\prime }=O\left[\left(N-PD\right)\left(\left(\eta_{9}+\eta_{6}\right)D+f\left(D\right)\right)\right]=O\left[D+f\left(D\right)\right]\text{.} \end{array}$$

The time complexity *T*_3_′ of updating the position of scouters is the same as equation (24).

To sum up, the time complexity of ISSA is

(29) $$\begin{array}{c} T^{\prime }={T_{0}}^{\prime }+T_{\max}\left({T_{1}}^{\prime }+{T_{2}}^{\prime }+{T_{3}}^{\prime }\right)=O\left[D+f\left(D\right)\right]\text{.} \end{array}$$

### 5.5. Performance of the ISSA in HIFU Sound Field Optimization

The ISSA is used to optimize HIFU sound field to test its performance in practical engineering problems. Without loss of generality, the optimization effects of ISSA under symmetric focal point and asymmetric focal point are investigated, respectively.

**Figure 4:**
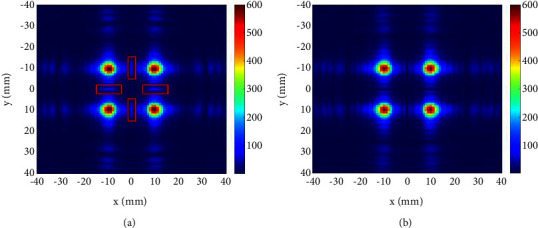
Sound field distribution on *z* = 100 mm plane with the symmetric focal point: (a) unoptimized and (b) ISSA-optimized.

**Figure 5:**
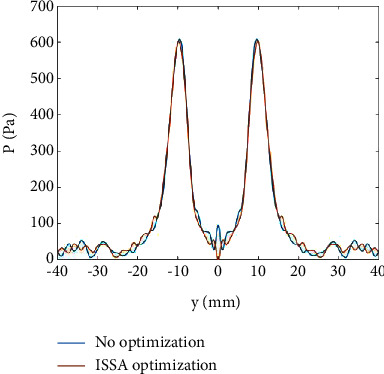
Variation curve of sound pressure *P* with *y* on the axis of *x* = −10 mm.

In the case of symmetric focal point, we set four focal points, whose coordinates in the *z* direction are 100*mm*, and their coordinates in the *xy* plane are (10,10)*mm*, (−10,10)*mm*, (−10, −10)*mm*, and (10, −10)*mm*, respectively. The distribution of unoptimized sound field and the ISSA-optimized sound field on the *z*=100*mm* plane is shown in Figure 4. On the axis of *x*=−10*mm*, the variation curve of sound pressure *P* with *y* is shown in Figure 5.

As shown in Figure 4, in the unoptimized sound field, there are obvious acoustic sidelobes between two adjacent focal points (marked with red rectangle box in Figure 4), and its amplitude is 94.5 Pa. After ISSA optimization, these acoustic sidelobes have been suppressed. The amplitude is weakened to 1.1866*E* − 05 Pa, and the energy of the acoustic wave is more concentrated to the focal points. It can be seen from the calculation that the percentage of sound pressure improvement is nearly 100%. This improvement can be more clearly observed in Figure 5. In Figure 5, the two curves have a very clear difference around *y*=0*mm*, and the blue curve is always above the red curve, which means that the acoustic sidelobes are very obvious in the unoptimized sound field, while after ISSA optimization, the acoustic sidelobes in the sound field have been suppressed. Here, the focal point sound pressure is set to 600 Pa, and the acoustic sidelobe sound pressure has reached 94.5 Pa, accounting for 15.75% of the focal point. It can be seen that there is a lot of waste of sound energy. It is very necessary to suppress the acoustic sidelobe and improve the focal point energy.

**Figure 6:**
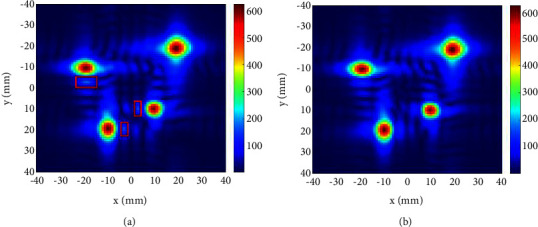
Sound field distribution on *z* = 200 mm plane with the asymmetric focal point: (a) unoptimized and (b) ISSA-optimized.

**Figure 7:**
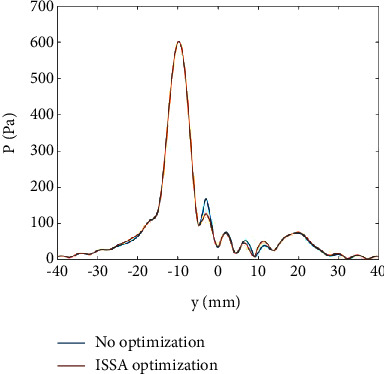
Variation curve of sound pressure *P* with *y* on the axis of *x* = −20 mm.

In the case of the asymmetric focal point, we also set four focal points. Their coordinates in the *z* direction are 200*mm*, and their coordinates in the *xy* plane are (10,10)*mm*, (−10,20)*mm*, (−20, −10)*mm*, and (20, −20)*mm*, respectively. The distribution of the unoptimized sound field and the ISSA-optimized sound field on the *z*=200*mm* plane is shown in Figure 6. On the axis of *x*=−20*mm*, the variation curve of sound pressure *P* with *y* is shown in Figure 7.

As shown in Figure 6, in the unoptimized sound field, there are three obvious acoustic sidelobes (marked with a red rectangle box in Figure 6), and their amplitudes from top to bottom are 168.11 Pa, 163.43 Pa, and 157.43 Pa. After ISSA optimization, these acoustic sidelobes are suppressed to different degrees, and their amplitudes are weakened to 116.05 Pa, 108.67 Pa, and 121.51 Pa, respectively. The percentage of sound pressure improvement is 30.97%, 33.51%, and 30.97%, respectively. This improvement can be more clearly observed in Figure 7. In Figure 7, the amplitude of the acoustic sidelobe near *y*=0*mm* before optimization is 168.11 Pa, accounting for 28.02% of the focal point. After ISSA optimization, the acoustic sidelobe is weakened to 116.05 Pa, accounting for 19.34% of the focal point. The improvement is about 10%. The energy of the sound field is more concentrated after ISSA optimization, which is very beneficial for HIFU treatment.

## 6. Conclusions

This paper presents an improved sparrow search algorithm, which overcomes some shortcomings of SSA and improves the convergence performance and stability of SSA [32]. ISSA uses Chebyshev chaotic map and elite opposition-based learning strategy to initialize the population and improve the quality of the initial population. The dynamic weight factor and Levy flight strategy are introduced into the position update equation of producers to avoid falling into the local optimal solution. The mutation strategy is introduced into the position update equation of scroungers to increase the diversity of the population. In order to verify the feasibility and effectiveness of ISSA, the performance of ISSA on 23 benchmark functions is compared with that of the GA, PSO, GWO, WOA, HHO, SSA, and SNS. The results show that ISSA is superior to the other seven algorithms in convergence speed, convergence accuracy, and stability. In order to test the performance of ISSA in practical engineering problems, ISSA is used for HIFU sound field optimization. The results show that ISSA can effectively suppress the acoustic sidelobe and improve the focusing ability of sound waves, which is of great benefit for HIFU treatment. The significance of this paper is as follows:

- Improve the sparrow search algorithm, enhance the quality of the initial population, and the ability to jump out of the local optimal solution
- Establish a 256-element concave spherical phased array transducer model, use ISSA to optimize the HIFU sound field, effectively suppress the acoustic sidelobe, improve the focusing performance, and provide a new idea for the research of HIFU technology

In future work, we will further optimize ISSA and use it to solve other engineering problems. At the same time, we will also pay attention to other advanced optimization algorithms and make further research.

## Figures and Tables

**Algorithm 1:**
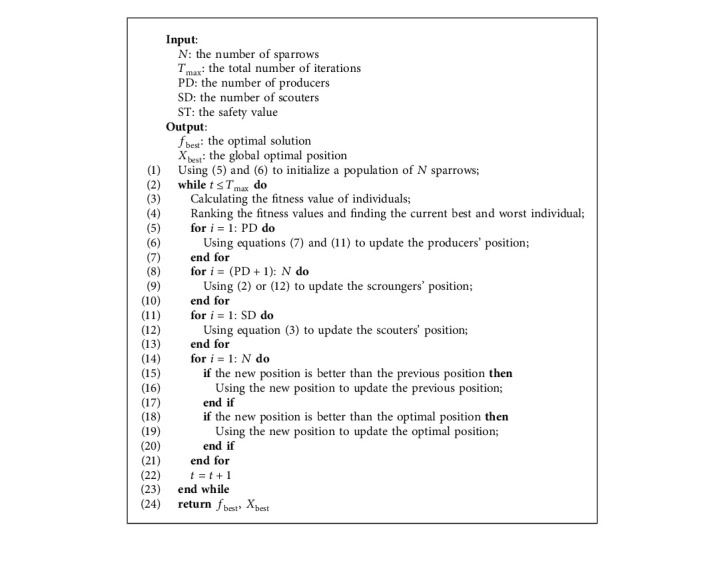
The improved sparrow search algorithm.

**Table 1:** Unimodal functions (dim = 30).

Functions	Range	*F*_min_
*F*_1_(*x*)=∑_*i*=1_^*n*^*x*_*i*_^2^	[−100, 100]	0
$F_{2}\left(x\right)=\sum_{i=1}^{n}\left|x_{i}\right|+\overset{n}{\underset{i=1}{\prod }}\left|x_{i}\right|$	[−10, 10]	0
*F*_3_(*x*)=∑_*i*=1_^*n*^(∑_*j*=1_^*i*^*x*_*j*_)^2^	[−100, 100]	0
*F*_4_(*x*)=max {|*x*_*i*_|, 1 ≤ *i* ≤ *n*}	[−100, 100]	0
*F*_5_(*x*)=∑_*i*=1_^*n*−1^[100(*x*_*i*+1_ − *x*_*i*_^2^)^2^+(*x*_*i*_ − 1)^2^]	[−30, 30]	0
*F*_6_(*x*)=∑_*i*=1_^*n*−1^(*x*_*i*_+0.5)^2^	[−100, 100]	0
*F*_7_(*x*)=∑_*i*=1_^*n*^*ix*_*i*_^4^+rando m(0,1)	[−1.28, 1.28]	0

**Table 2:** Multimodal functions (dim = 30).

Functions	Range	*F*_min_
$F_{8}\left(x\right)=\sum_{i=1}^{n}-x_{i}\sin\left(\sqrt{\left|x_{i}\right|}\right)$	[−500, 500]	−418.9829 n
*F*_9_(*x*)=∑_*i*=1_^*n*^[*x*_*i*_^2^ − 10 cos (2*πx*_*i*_)+10]	[−5.12, 5.12]	0
$F_{10}\left(x\right)=-20\;\exp\;\left(-0.2\sqrt{1/n\sum_{i=1}^{n}{x_{i}}^{2}}\right)-\exp\;\left(1/n\sum_{i=1}^{n}\cos\;\left(2\pi x_{i}\right)\right)+20+e$	[−32, 32]	0
$F_{11}\left(x\right)=1/4000\sum_{i=1}^{n-1}{x_{i}}^{2}-\overset{n}{\underset{i=1}{\prod }}\cos\;\left(x_{i}/\sqrt{i}\right)+1$	[−600, 600]	0
*F*_12_(*x*)=*π*/*n*{10 sin^2^ (*πy*_1_)+∑_*i*=1_^*n*−1^(*y*_*i*_ − 1)^2^[1+10 sin^2^ (*πy*_*i*+1_)]+(*y*_*n*_ − 1)^2^}	[−50, 50]	0
+∑_*i*=1_^*n*^*u*(*x*_*i*_, 10,100,4)*y*_*i*_=1+(*x*_*i*_+1)/4		
$u\left(x_{i},a,k,m\right)=\left\{\begin{array}{cc} k{\left(x_{i}-a\right)}^{m} & x_{i}>a\\ 0 & -a<x_{i}<a\\ k{\left(-x_{i}-a\right)}^{m} & x_{i}<-a \end{array}\right.$		
*F*_13_(*x*)=0.1{ sin^2^ (3*πx*_1_)+∑_*i*=1_^*n*−1^(*x*_*i*_ − 1)^2^[1+sin^2^ (3*πx*_*i*+1_)]+(*x*_*n*_ − 1)^2^·[1+sin^2^ (2*πx*_*n*_)]}+∑_*i*=1_^*n*^*u*(*x*_*i*_, 5,100,4)	[−50, 50]	0

**Table 3:** Fixed-dimension functions.

Functions	Dim	Range	*F*_min_
*F*_14_(*x*)=(1/500+∑_*j*=1_^25^1/(*j*+(*x*_1_ − *a*_1*j*_)^6^+(*x*_2_ − *a*_2*j*_)^6^)	2	[−65, 65]	1
*F*_15_(*x*)=∑_*i*=1_^11^[*a*_*i*_ − (*x*_1_(*b*_*i*_^2^+*b*_*i*_*x*_2_))/(*b*_*i*_^2^+*b*_*i*_*x*_3_+*x*_4_)]^2^	4	[−5, 5]	0.0003
*F*_16_(*x*)=4*x*_1_^2^ − 2.1*x*_1_^4^+1/3*x*_1_^6^+*x*_1_*x*_2_ − 4*x*_2_^2^+4*x*_2_^4^	2	[−5, 5]	−1.0316
*F*_17_(*x*)=(*x*_2_ − 5.1/4*π*^2^*x*_1_^2^+5/*πx*_1_ − 6)^2^+10(1 − 1/8*π*)cos*x*_1_+10	2	[−5, 5]	0.398
*F*_18_(*x*)=[1+(*x*_1_+*x*_2_+1)^2^(19 − 14*x*_1_+3*x*_1_^2^ − 14*x*_2_+6*x*_1_*x*_2_+3*x*_2_^2^)] · [30+(2*x*_1_ − 3*x*_2_)^2^(18 − 32*x*_1_+12*x*_1_^2^+48*x*_2_ − 36*x*_1_*x*_2_+27*x*_2_^2^)]	2	[−2, 2]	3
*F*_19_(*x*)=−∑_*i*=1_^4^*c*_*i*_exp [∑_*j*=1_^3^*a*_*ij*_(*x*_*j*_ − *p*_*ij*_)^2^]	3	[0, 1]	−3.86
*F*_20_(*x*)=−∑_*i*=1_^4^*c*_*i*_exp [∑_*j*=1_^6^*a*_*ij*_(*x*_*j*_ − *p*_*ij*_)^2^]	6	[0, 1]	−3.32
*F*_21_(*x*)=−∑_*i*=1_^5^[(*X* − *α*_*i*_)(*X* − *α*_*i*_)^*T*^+*c*_*i*_]^−1^	4	[0, 10]	−10.1532
*F*_22_(*x*)=−∑_*i*=1_^7^[(*X* − *α*_*i*_)(*X* − *α*_*i*_)^*T*^+*c*_*i*_]^−1^	4	[0, 10]	−10.4028
*F*_23_(*x*)=−∑_*i*=1_^10^[(*X* − *α*_*i*_)(*X* − *α*_*i*_)^*T*^+*c*_*i*_]^−1^	4	[0, 10]	−10.5363

**Table 4:** Minimum, average, and standard deviation of eight algorithms on benchmark functions.

Func	Algorithm	*F*_min_	Avg	Std
F1	GA	0.1726	0.3688	0.1058
	PSO	0.1152	0.3618	0.2198
	GWO	7.4055*E* − 43	4.2803*E* − 41	8.5127*E* − 41
	WOA	2.4924*E* − 105	9.1573*E* − 97	2.7034*E* − 96
	HHO	5.0860*E* − 120	3.6083*E* − 106	1.3799*E* − 105
	SSA	0	0	0
	ISSA	0	0	0
	SNS	4.5158*E* − 80	3.3939*E* − 78	5.7930*E* − 78

F2	GA	1.3102	2.2009	0.3276
	PSO	1.3232	3.1762	1.3121
	GWO	7.9563*E* − 25	5.6060*E* − 24	4.0470*E* − 24
	WOA	1.5696*E* − 64	2.1231*E* − 57	7.3635*E* − 57
	HHO	2.2888*E* − 64	5.0350*E* − 56	1.4616*E* − 55
	SSA	0	5.9657*E* − 246	0
	ISSA	0	0	0
	SNS	1.1772*E* − 41	1.2192*E* − 40	8.8551*E* − 41

F3	GA	35.0637	113.5643	98.2013
	PSO	9.3528	31.9008	17.2877
	GWO	1.0519*E* − 14	1.7352*E* − 11	6.7481*E* − 11
	WOA	3.0453*E* + 03	1.3447*E* + 04	6.1948*E* + 03
	HHO	1.9320*E* − 112	1.9745*E* − 95	4.6188*E* − 95
	SSA	0	1.6209*E* − 303	0
	ISSA	0	0	0
	SNS	4.2048*E* − 30	2.8538*E* − 23	7.9304*E* − 23

F4	GA	0.8578	4.0991	2.0845
	PSO	1.0475	5.0234	2.7751
	GWO	1.5166*E* − 11	1.4806*E* − 10	1.1322*E* − 10
	WOA	6.8169*E* − 12	20.9694	23.8119
	HHO	8.1970*E* − 59	1.4348*E* − 54	4.9994*E* − 54
	SSA	0	9.1203*E* − 237	0
	ISSA	0	0	0
	SNS	3.1845*E* − 36	1.5309*E* − 35	1.2401*E* − 35

F5	GA	132.8354	328.3109	172.5915
	PSO	46.0601	244.4676	309.2162
	GWO	24.9588	26.1575	0.7470
	WOA	26.2030	26.8192	0.2945
	HHO	3.2743*E* − 05	0.0012	0.0016
	SSA	9.8449*E* − 09	1.2272*E* − 05	2.8983*E* − 05
	ISSA	2.5419*E* − 09	7.6049*E* − 09	1.3492*E* − 05
	SNS	25.2683	26.1652	0.3588

F6	GA	0.2511	0.4112	0.1158
	PSO	0.0927	0.3191	0.1521
	GWO	1.5281*E* − 05	6.3023*E* − 05	0.1987
	WOA	0.0023	0.0043	0.0017
	HHO	5.8671*E* − 08	7.3739*E* − 06	1.1985*E* − 05
	SSA	6.6045*E* − 11	7.0626*E* − 07	1.0864*E* − 07
	ISSA	3.9263*E* − 13	4.8787*E* − 11	7.7172*E* − 10
	SNS	4.0101*E* − 07	1.4531*E* − 05	2.8176*E* − 05

F7	GA	0.4491	0.8592	0.3148
	PSO	0.1019	0.2901	0.1288
	GWO	1.6605*E* − 04	1.0398*E* − 03	2.6688*E* − 04
	WOA	6.5593*E* − 05	5.2027*E* − 04	8.3366*E* − 03
	HHO	2.7958*E* − 06	3.7936*E* − 06	3.9055*E* − 05
	SSA	8.5655*E* − 06	1.0812*E* − 04	8.7271*E* − 05
	ISSA	9.4541*E* − 07	6.7345*E* − 06	5.4023*E* − 05
	SNS	2.8520*E* − 05	4.8744*E* − 05	1.0430*E* − 04

F8	GA	−1.8296*E* + 03	−1.4652*E* + 03	170.2547
	PSO	−8.6206*E* + 03	−8.1473*E* + 03	764.9701
	GWO	−8.5730*E* + 03	−6.7798*E* + 03	543.8278
	WOA	−1.2569*E* + 04	−1.2524*E* + 04	1.2405*E* + 03
	HHO	−1.2621*E* + 04	−1.2569*E* + 04	627.8604
	SSA	−9.6550*E* + 03	−9.2471*E* + 03	661.9266
	ISSA	−1.2569*E* + 04	−1.1637*E* + 04	1.9303*E* + 03
	SNS	−9.1063*E* + 03	−8.5178*E* + 03	274.8565

F9	GA	22.7386	43.8443	18.1360
	PSO	35.6856	63.8049	15.2725
	GWO	0	157.723	2.8713
	WOA	0	0	0
	HHO	0	0	0
	SSA	0	0	0
	ISSA	0	0	0
	SNS	0	0	0

F10	GA	0.5610	1.9370	1.2596
	PSO	1.7736	4.5744	3.1795
	GWO	2.2204*E* − 14	2.8362*E* − 14	3.3553*E* − 15
	WOA	8.8818*E* − 16	8.8818*E* − 16	2.4567*E* − 15
	HHO	8.8818*E* − 16	8.8818*E* − 16	0
	SSA	8.8818*E* − 16	8.8818*E* − 16	0
	ISSA	8.8818*E* − 16	8.8818*E* − 16	0
	SNS	8.8818*E* − 16	8.8818*E* − 16	0

F11	GA	0.0110	0.0803	0.0507
	PSO	0.4939	1.3628	0.8000
	GWO	0	0	0
	WOA	0	0	0
	HHO	0	0	0
	SSA	0	0	0
	ISSA	0	0	0
	SNS	0	0	0

F12	GA	0.0017	0.0083	0.0157
	PSO	5.4913	13.3800	4.7394
	GWO	1.3595*E* − 06	0.0167	0.0133
	WOA	1.6101*E* − 04	0.0013	0.0024
	HHO	3.5147*E* − 08	1.1030*E* − 06	1.3409*E* − 06
	SSA	3.6602*E* − 13	4.4157E-11	1.1270*E* − 08
	ISSA	5.4643*E* − 17	1.9518*E* − 13	1.2207*E* − 11
	SNS	1.8399*E* − 08	5.5631*E* − 08	8.9952*E* − 08

F13	GA	0.0367	0.0882	0.0265
	PSO	0.0637	0.4392	2.0113
	GWO	1.4168*E* − 05	0.1006	0.1266
	WOA	0.0055	0.0068	0.0287
	HHO	2.5137*E* − 08	4.0881*E* − 06	1.2922*E* − 05
	SSA	4.5725*E* − 11	2.7827*E* − 09	1.1367*E* − 07
	ISSA	1.7253*E* − 14	1.3480*E* − 12	3.0559*E* − 10
	SNS	4.7620*E* − 07	0.0110	0.0401

F14	GA	0.9980	0.9980	1.1822*E* − 05
	PSO	0.9980	2.9821	1.2826
	GWO	0.9980	1.1206	1.9074
	WOA	0.9980	1.1641	0.3622
	HHO	0.9980	0.9983	3.4100*E* − 11
	SSA	0.9980	14.7720	16.9265
	ISSA	0.9980	1.1084	1.9682
	SNS	0.9980	0.9980	0

F15	GA	9.2281*E* − 04	1.1875*E* − 03	4.9309*E* − 04
	PSO	3.0749*E* − 04	1.2237*E* − 03	3.8690*E* − 04
	GWO	3.0749*E* − 04	5.0112*E* − 04	6.8127*E* − 04
	WOA	3.0772*E* − 04	7.1811*E* − 04	4.3624*E* − 04
	HHO	3.0749*E* − 04	5.3561*E* − 04	2.3027*E* − 04
	SSA	3.0749*E* − 04	3.0801*E* − 04	8.1578*E* − 07
	ISSA	3.0749*E* − 04	3.0749*E* − 04	1.6916*E* − 08
	SNS	3.0749*E* − 04	3.0921*E* − 04	1.0241*E* − 05

F16	GA	−1.0308	−1.0093	0.0269
	PSO	−1.0316	−1.0314	6.7752*E* − 15
	GWO	−1.0316	−1.0315	1.4321*E* − 05
	WOA	−1.0316	−1.0315	1.5771*E* − 11
	HHO	−1.0316	−1.0316	3.5948*E* − 14
	SSA	−1.0316	−1.0315	6.6486*E* − 15
	ISSA	−1.0316	−1.0316	6.4539*E* − 16
	SNS	−1.0316	−1.0315	6.7752*E* − 15

F17	GA	0.3979	0.4099	0.0154
	PSO	0.3979	0.3979	0
	GWO	0.3979	0.3979	3.4787*E* − 07
	WOA	0.3979	0.3979	9.4224*E* − 08
	HHO	0.3979	0.3979	5.5899*E* − 10
	SSA	0.3979	0.3979	0
	ISSA	0.3979	0.3979	0
	SNS	0.3979	0.3979	0

F18	GA	3.0191	4.2707	20.0975
	PSO	3.0000	3.0000	1.9110*E* − 15
	GWO	3.0000	3.0000	4.5137*E* − 10
	WOA	3.0000	3.0000	7.6160*E* − 06
	HHO	3.0000	3.0000	1.0883*E* − 10
	SSA	3.0000	3.0000	2.0748*E* − 14
	ISSA	3.0000	3.0000	1.1778*E* − 15
	SNS	3.0000	3.0000	2.2296*E* − 14

F19	GA	−3.8125	−1.7296	1.4953
	PSO	−3.8244	−3.8244	1.3550*E* − 15
	GWO	−3.8244	−3.8244	1.2697*E* − 06
	WOA	−3.8244	−3.8244	3.3715*E* − 04
	HHO	−3.8244	−3.8243	1.8163*E* − 04
	SSA	−3.8244	−3.8244	1.3550*E* − 15
	ISSA	−3.8244	−3.8244	1.3550*E* − 15
	SNS	−3.8244	−3.8244	1.3550*E* − 15

F20	GA	−3.2285	−2.3482	0.9614
	PSO	−3.3220	−3.2625	0.0605
	GWO	−3.3220	−3.2590	0.0711
	WOA	−3.3220	−3.2532	0.0798
	HHO	−3.3179	−3.2014	0.0784
	SSA	−3.3220	−3.2507	0.0592
	ISSA	−3.3220	−3.2744	0.0592
	SNS	−3.3220	−3.2520	0.0624

F21	GA	−9.8954	−4.4387	4.6735
	PSO	−10.1532	−2.6828	3.4455
	GWO	−10.1531	−5.0415	1.7986
	WOA	−10.1532	−9.9830	0.9307
	HHO	−10.1500	−5.0550	1.2921
	SSA	−10.1532	−10.1531	1.5634*E* − 04
	ISSA	−10.1532	−9.5262	1.4992
	SNS	−10.1532	−10.1531	7.1207*E* − 05

F22	GA	−10.2468	−3.3236	6.0573
	PSO	−10.4029	−10.3015	1.5411
	GWO	−10.4029	−10.0491	1.3432
	WOA	−10.4029	−10.0132	1.3732
	HHO	−10.3999	−5.7947	1.8339
	SSA	−10.4029	−10.4028	4.0757*E* − 03
	ISSA	−10.4029	−10.4027	2.0212*E* − 05
	SNS	−10.4029	−10.3021	1.1427*E* − 03

F23	GA	−10.4686	−2.0108	3.1604
	PSO	−10.5364	−10.5181	0.6198
	GWO	−10.5363	−5.1064	1.7620
	WOA	−10.5364	−9.1405	2.6164
	HHO	−10.5358	−5.1278	1.8662
	SSA	−10.5364	−10.5363	1.6540*E* − 04
	ISSA	−10.5364	−10.3659	2.5198*E* − 04
	SNS	−10.5364	−10.3445	1.8067*E* − 03

**Table 5:** Experimental results of different improvement strategies.

Func	Algorithm	*F *_min_	Avg	Std
F2	SSA	0	1.6886*E* − 318	0
	SSA01	0	0	0
	SSA02	0	0	0
	SSA03	0	0	0
	ISSA	0	0	0

F5	SSA	4.0723*E* − 09	4.2421*E* − 05	1.5023*E* − 05
	SSA01	3.5211*E* − 09	3.8830*E* − 07	1.1986*E* − 05
	SSA02	3.0467*E* − 09	1.4628*E* − 06	5.3375*E* − 06
	SSA03	3.4656*E* − 09	1.3116*E* − 06	4.2906*E* − 06
	ISSA	1.0824*E* − 09	9.7182*E* − 08	2.8898*E* − 06

F7	SSA	3.5673*E* − 06	1.6544*E* − 04	1.0126*E* − 04
	SSA01	1.4294*E* − 06	1.1571*E* − 04	9.2207*E* − 05
	SSA02	3.3467*E* − 06	6.7627*E* − 05	7.4811*E* − 05
	SSA03	3.0225*E* − 06	1.5858*E* − 04	7.9233*E* − 05
	ISSA	1.3805*E* − 06	4.4199*E* − 05	7.3731*E* − 05

F9	SSA	0	0	0
	SSA01	0	0	0
	SSA02	0	0	0
	SSA03	0	0	0
	ISSA	0	0	0

F12	SSA	2.8537*E* − 13	1.1162*E* − 11	2.8143*E* − 09
	SSA01	1.8369*E* − 15	3.3307*E* − 13	9.0648*E* − 11
	SSA02	3.0190*E* − 15	7.2377*E* − 12	1.9612*E* − 10
	SSA03	3.5533*E* − 16	5.3059*E* − 15	1.8971*E* − 10
	ISSA	2.9885*E* − 16	2.0635*E* − 13	4.9317*E* − 11

F13	SSA	2.0502*E* − 12	7.3101*E* − 10	1.3270*E* − 09
	SSA01	1.8026*E* − 14	5.9631*E* − 14	2.5349*E* − 10
	SSA02	7.8553*E* − 13	1.7663*E* − 10	2.5502*E* − 10
	SSA03	3.1532*E* − 14	4.6639*E* − 11	2.0181*E* − 10
	ISSA	1.1261*E* − 14	3.1549*E* − 14	1.6795*E* − 10

F14	SSA	0.9980	10.7632	7.2905
	SSA01	0.9980	1.1187	2.7074
	SSA02	0.9980	1.1158	2.8284
	SSA03	0.9980	1.2208	2.7056
	ISSA	0.9980	1.1002	2.1311

F21	SSA	−10.1532	−10.1531	6.1446*E* − 05
	SSA01	−10.1532	−10.1532	9.2352*E* − 04
	SSA02	−10.1532	−10.1532	1.7586
	SSA03	−10.1532	−10.1532	5.9467*E* − 05
	ISSA	−10.1532	−10.1532	0.9302

**Table 6:** Wilcoxon's rank-sum test.

Functions	GA		PSO		GWO		WOA		HHO		SSA		SNS	
	*P*	*R*	*p*	*R*	*p*	*R*	*p*	*R*	*p*	*R*	*p*	*R*	*p*	*R*
F1	1.2118*E* − 12	+	1.2118*E* − 12	+	1.2118*E* − 12	+	1.2118*E* − 12	+	1.2118*E* − 12	+	NAN	=	1.2118*E* − 12	+
F2	1.2118*E* − 12	+	1.2118*E* − 12	+	1.2118*E* − 12	+	1.2118*E* − 12	+	1.2118*E* − 12	+	1.4552*E* − 04	+	1.2118*E* − 12	+
F3	1.2118*E* − 12	+	1.2118*E* − 12	+	1.2118*E* − 12	+	1.2118*E* − 12	+	1.2118*E* − 12	+	0.0815	−	1.2118*E* − 12	+
F4	1.2118*E* − 12	+	1.2118*E* − 12	+	1.2118*E* − 12	+	1.2118*E* − 12	+	1.2118*E* − 12	+	0.0014	+	1.2118*E* − 12	+
F5	3.0199*E* − 11	+	3.0199*E* − 11	+	3.0199*E* − 11	+	3.0199*E* − 11	+	6.7220*E* − 10	+	0.0408	+	3.0199*E* − 11	+
F6	3.0199*E* − 11	+	3.0199*E* − 11	+	3.0199*E* − 11	+	3.0199*E* − 11	+	3.3384*E* − 11	+	4.1997*E* − 10	+	3.0199*E* − 11	+
F7	3.0199*E* − 11	+	3.0199*E* − 11	+	8.9934*E* − 11	+	4.1997*E* − 10	+	2.7086*E* − 03	+	6.7650*E* − 05	+	1.1023*E* − 08	+
F8	3.0199*E* − 11	+	5.9673*E* − 09	+	3.8202*E* − 10	+	1.0261*E* − 03	+	0.0033	+	2.5721*E* − 07	+	6.5277*E* − 08	+
F9	1.2118*E* − 12	+	1.2118*E* − 12	+	1.1022*E* − 03	+	NAN	=	NAN	=	NAN	=	NAN	=
F10	1.2118*E* − 12	+	1.2118*E* − 12	+	6.3567*E* − 10	+	3.8580*E* − 07	+	NAN	=	NAN	=	NAN	=
F11	1.2118*E* − 12	+	1.2118*E* − 12	+	NAN	=	NAN	=	NAN	=	NAN	=	NAN	=
F12	3.0199*E* − 11	+	3.0199*E* − 11	+	3.0199*E* − 11	+	3.0199*E* − 11	+	3.0199*E* − 11	+	3.4971*E* − 09	+	3.0199*E* − 11	+
F13	3.0199*E* − 11	+	3.0199*E* − 11	+	3.0199*E* − 11	+	3.0199*E* − 11	+	3.0199*E* − 11	+	1.4643*E* − 10	+	3.0199*E* − 11	+
F14	5.8389*E* − 03	+	1.6289*E* − 05	+	5.1425*E* − 04	+	5.8375*E* − 04	+	3.7142*E* − 03	+	1.2118*E* − 12	+	8.5920*E* − 07	+
F15	3.0199*E* − 11	+	9.3096*E* − 06	+	3.4742*E* − 10	+	3.0199*E* − 11	+	3.0199*E* − 11	+	4.0772*E* − 06	+	2.1540*E* − 08	+
F16	1.7203*E* − 12	+	0.0026	+	1.7203*E* − 12	+	1.7189*E* − 12	+	9.3658*E* − 03	+	0.0122	+	0.0027	+
F17	1.2118*E* − 12	+	NAN	=	1.2118*E* − 12	+	1.2118*E* − 12	+	4.5664*E* − 12	+	NAN	=	NAN	=
F18	1.9356*E* − 09	+	0.0474	+	7.1021*E* − 08	+	7.1021*E* − 08	+	8.6673*E* − 07	+	0.0321	+	0.0210	+
F19	1.2118*E* − 12	+	NAN	=	1.2118*E* − 12	+	1.2118*E* − 12	+	1.2118*E* − 12	+	NAN	=	NAN	=
F20	4.9074*E* − 09	+	0.0277	+	0.0017	+	0.0017	+	3.2156*E* − 04	+	3.2156*E* − 04	+	0.0017	+
F21	1.2828*E* − 10	+	4.6208*E* − 06	+	4.2895*E* − 05	+	6.1378*E* − 04	+	3.3591*E* − 10	+	2.1246*E* − 09	+	1.8400*E* − 09	+
F22	1.1208*E* − 10	+	9.9211*E* − 10	+	2.0048*E* − 04	+	4.0546*E* − 05	+	1.8240*E* − 10	+	4.9973*E* − 04	+	7.6337*E* − 06	+
F23	2.7604*E* − 09	+	0.0082	+	0.0077	+	5.5777*E* − 04	+	2.4325*E* − 10	+	0.0077	+	1.4179*E* − 03	+

## Data Availability

Part of the data supporting the results of this study is available from the corresponding authors.
